# Immediate effects of passive stretching and/or local vibration on ankle range of motion, calf muscle stiffness and passive torque: a randomized controlled cross-over trial

**DOI:** 10.1007/s00421-025-05839-6

**Published:** 2025-06-12

**Authors:** Daniel Jochum, Andreas Konrad, Josef Fischer, Stanislav D. Siegel, Konstantin Warneke

**Affiliations:** 1 Department of Health Science and Technology, ETH Zürich, Zurich, Switzerland; 2 Institute of Human Movement Science, Sport and Health, University of Graz, Graz, Austria; 3 Department of Human Movement Science and Exercise Physiology, Friedrich Schiller University Jena, Jena, Germany

**Keywords:** Vibratory stimulation, Flexibility, Ultrasound, Dynamometry

## Abstract

**Purpose:**

Vibrational stimulation was suggested sufficient to acutely enhance range of motion (ROM). However, the actual merit of superimposed vibration and underlying mechanical or sensory mechanisms of ROM increases due to local vibration are not well understood.

**Methods:**

Using a randomized controlled cross-over trial, this study opposed two minutes of stretching + vibration (STV) to vibration (V) and stretching (ST) alone as well as a passive control (CG) in 30 healthy, recreationally active participants. Pre- and post-intervention measurements of ankle dorsiflexion ROM, ankle passive torque, and gastrocnemius medialis muscle stiffness were conducted using a dynamometer and shear wave elastography, respectively.

**Results:**

All interventions significantly increased ROM (*p* < 0.001, *η*^2^ = 0.622) compared to CG, with no additional benefits observed for vibration combined with stretching compared to stretching or vibration alone. However, combining stretching and vibration produced the most pronounced reductions in passive torque over the whole ankle ROM. The combination demonstrated additive effects of stretching and vibration, which affected passive torque in the end ROM and at rest, respectively. Muscle stiffness decreased, but did not differentiate between conditions.

**Conclusion:**

These findings negate additional acute vibration effects on ROM that surpass the effects of stretching alone, while the combination of stretching and vibration presented the most prominent influence on passive torque and joint resistance. Vibration also increases stretching pain tolerance; this might facilitate the conduction of exercises and improves compliance as well as adherence to stretching habits.

**Supplementary Information:**

The online version contains supplementary material available at 10.1007/s00421-025-05839-6.

## Introduction

Flexibility is, besides strength and endurance, considered of fundamental importance in sports, health and rehabilitation settings (Garber et al. 2011). Reaching higher range of motion (ROM) is often associated with increased performance and some articles propose reduced injury risk (Bradley and Portas 2007; Wilk et al. 2015). Consequently, it is not surprising that there are hundreds of articles seeking to develop novel and more effective exercise routines to optimize ROM training programs. In this regard, stretching might be one of the most common interventions chosen to enhance ROM acutely (Behm et al. 2023), and chronically (Konrad et al. 2023). While different types of stretching as a stand-alone intervention were effective in increasing ROM, evidence suggests accumulated effects when superimposing additional contractions in high muscle lengths (Mizuno 2019, 2023). While, for instance, proprioceptive neuromuscular facilitation (PNF) requires an active muscle activation of the stretching agonist or the shortened antagonist, additive muscle activity can be also induced passively.

One such opportunity is provided by inducing vibration stimuli. Studies commonly refer to contractions induced via the tonic vibration reflex (TVR) (Hagbarth and Eklund 1966), which can be considered as a reaction of the muscle–tendon unit to dampen the vibratory waves caused by the vibration device. In the literature, additive positive effects of vibration training were attributed to neural (Fowler et al. 2019), pain sensory (Casale and Hansson 2022), circulatory and thermoregulatory effects (Fuller et al. 2013) while also mechano-chemical signal conversion (Lewis et al. 2017) is discussed. Nevertheless, the practical implementation and validation of these theoretical mechanisms remain underexplored in current research. Several studies investigating the effects of combined stretching and vibration on ROM (Jochum et al. 2025a), but few articles reported results for underlying/associated parameters, such as passive torque and/or stiffness (Rodrigues et al. 2017).

General vibration against passive controls has shown to be effective in increasing ROM acutely (Jochum et al. 2025a), but results are ambivalent regarding potential underlying mechanisms: A recent study of whole-body vibration (WBV) with different frequencies suggested that only the highest frequency of 24 Hz increased muscle stiffness with shear-wave speed (Muanjai et al. 2023). Other stiffness measurement methods including damped-oscillation techniques like myotonometry showed decreases in stiffness after percussive vibration treatments (Skinner et al. 2023). Passive peak torque at end ROM (PPT) was not affected by different local vibration frequencies (Pereira et al. 2020). Therefore, the influence of direct vibration seems to differ concerning the mode of application and might also be affected by the underlying exercise.

Studies combining vibration with stretching investigated a ratio between torque variation and ROM (Rodrigues et al. 2017) or myotonometry (Jochum et al. 2025b) found no differences between investigations and the control group. The current vibration evidence was summarized in a recent meta-analysis (Jochum et al. 2025a) calling for future research, as our understanding on vibration-induced effects is biased by meaningful heterogeneity and low-quality study designs, prohibiting evidence-based statements if vibration is a potential supplementation of implemented exercise applications.

The objective of this randomized controlled cross-over trial is to investigate the specific effects of two minutes of local vibration, both with and without stretching, compared to stretching alone and a control condition. Two minutes duration as it is a commonly used stretching duration demonstrated to acutely enhance flexibility and decrease muscle stiffness (Maeda et al. 2017; Konrad et al. 2017a, 2017b). The study evaluated the impact of the interventions on ankle range of motion (ROM), ankle passive torque over the whole ROM, and gastrocnemius medialis muscle stiffness, while also examining whether the combination of stretching and vibration results in cumulative benefits. It was hypothesized that the addition of local vibration with a high frequency (30 Hz) would further increase ROM and related parameters.

## Methods

### Experimental approach

**Fig. 1:**
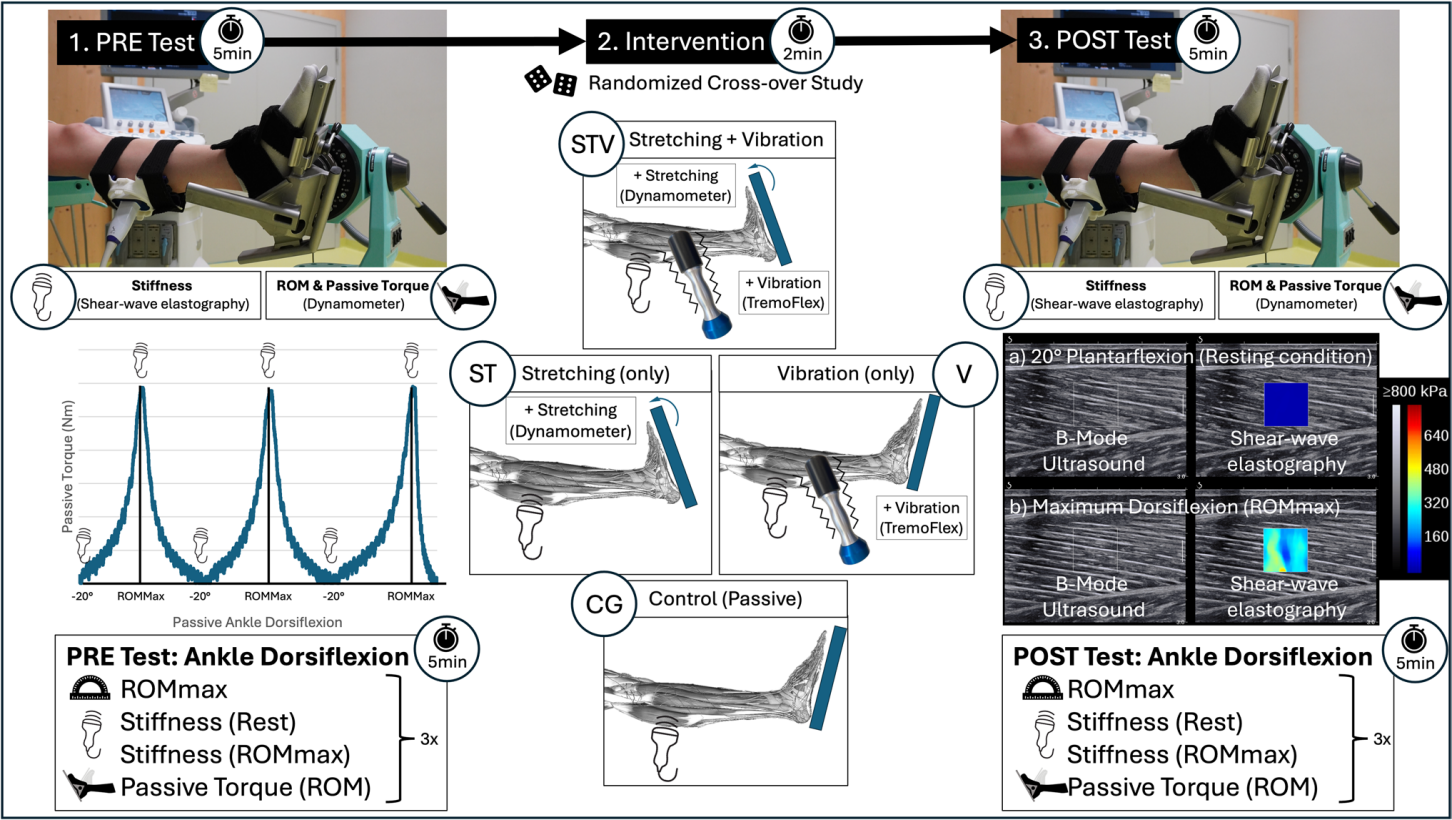
showing experimental procedure for assessment of ankle dorsiflexion range of motion (ROM), passive torque (dynamometer), and stiffness (shear-wave elastography) for before (1. PRE-Test) and after (3. POST-Test) the (2.) Interventions stretching with additional vibration (STV), stretching only (ST), vibration only (V), or passive control condition (CG) in randomized order in a repeated measures cross-over study

The study was designed as a randomized controlled cross-over trial to evaluate the acute effects of two minutes passive stretching with (STV) and without vibration (ST) and vibration only (V) against a passive control condition (CG) on flexibility. Testing and intervention were performed using the plantar flexors. ROM and related parameters such as passive torque at different ankle angle positions, stiffness in rest and maximal ROM were tested pre- and post-intervention in all 4 testing occasions (Fig. 1). All conditions were performed over an intervention period of maximal 10 days, with at least 48 h recovery between each session.

### Participants

Thirty healthy, recreationally active participants (16 males: age: 27.9 ± 5.4 years, height: 181.7 ± 6.2 cm, weight: 81.7 ± 12.3 kg/14 females: age: 26.7 ± 4.4 years, height: 166.4 ± 6.9 cm, weight: 61.9 ± 8.0 kg) were recruited from the University campus (Students in sports- and exercise science and physical education). They were classified recreationally active if they regularly and actively participated in an organized sports program and/or worked out in gyms at least twice per week. Exclusion criteria were any type of serious injury in the lower extremity within the last 6 months that required medical care (orthopaedic indication), varicose vains, thrombotic risk or any neurological disorder or pathology, including Parkinson disease, cerebral palsy and others. Furthermore, participants with electronic implants, osteoporosis, arteriosclerosis, prosthetics or pregnant women were not considered for inclusion. The study protocol was performed in adherence with the Declaration of Helsinki and all participants provided written informed consent for participation participant. The study protocol was approved by the local Ethical Committee of the University of Graz (GZ. 39/98/63 ex 2023/24).

### Measurements

Measurements were performed in the following order: (a) ROM assessment + (b) Shear-wave elastography (SWE) at 20° plantarflexion and dorsiflexion ROMmax; (c) Passive torque assessment in relation to ankle angle up to pre-determined ROMmax.

#### Dynamometry

An isokinetic dynamometer (Con-Trex Multijoint, CMV AG, Dübendorf, Switzerland) with the standard setup for ankle joint movements was used to assess ankle dorsiflexion ROM and passive resistive torque during passive dorsiflexion. Before testing started, participants performed a standardized and specific warm-up by moving the dynamometer over the full ROM. As soon as they were familiar with the device, testing started. The participants were positioned in a standardized sitting position at 110° hip flexion, while the machine was individually adjusted with the left foot (without wearing shoes) fixed with a strap to the foot plate and the knee extended to 180° (see Fig. 1). The position was chosen to decrease the length (Robinson and Probyn 2019) and, therefore, stiffness (Andrade et al. 2018) of the sciatic nerve. To ensure the same assessment position on the dynamometer in both appointments, the exact positions of the adapter and motor of the dynamometer were recorded after the first positioning. Participants were able to control the device with a remote control and to move their ankle passively from 20° ankle plantarflexion to the individual maximum ankle dorsiflexion. The approach was chosen to double-control the potential injury risk, while we assumed more accurate measurements, when participants could control the process. Since participants were not able to see reached ROM values the procedure was performed under blinded participants condition. This procedure was repeated for three (valid) times, and the maximum end ROM reached was used for the following passive torque assessment. Hereby, the ankle joint was moved passively for three cycles with a set angular velocity of 5°/s to avoid any reflexive muscle activity (Kubo et al. 2002; Mahieu et al. 2009; Konrad et al. 2017b). The participants were asked to relax during the procedure. During all tests, participants were kept unaware about their measured ankle angles, passive torque or muscle stiffness values.

#### Shear wave elastography

Muscle stiffness was determined via shear wave elastography (Aixplorer ultrasound scanner, version 11.0, Supersonic Imagine, Aix-en-Provence, France) on the gastrocnemius medialis. A linear transducer array (4–15 MHz, SuperLinear 15-4 Vermon, Tours, France) was coupled to the machine and used in SWE mode (musculoskeletal preset, penetration mode, smoothing level 5, persistence off, and scale 0–800 kPa).

To reach highest possible objectivity and reliability, the probe was fixed with a ProbeFix (Usono B.V., Eindhoven, Netherlands, https://www.usono.com/probefix-dynamic/) on the muscle belly of the target muscle. To account for individual calf muscle anatomy (short- and long plantar flexors), the distance between the muscle origin and attachment of the gastrocnemius medialis was determined via B-Mode Ultrasound and the thickest position was marked with a permanent marker to ensure that the investigation was performed at the same position on each occasion (4 testing days).

Three snapshots were taken in resting position (20° ankle plantarflexion) and at the maximum ROM position during pre- and post-measurements, respectively. Shear wave elastography mode incorporated a box that provided stiffness values (kPa), whereby the downscaled box was centred in the middle of the muscle.

### Interventions

After pre-testing all interventions were undertaken in the dynamometer. Therefore, the participants remained seated and fixed at 20° ankle plantarflexion for two minutes. For vibration (V) and control (CG), the ankle position remained in a 20° plantar flexed position. For stretching (S) and the combined stretching + vibration condition (STV), the calf muscle was passively stretched by moving the dynamometer so that the foot was positioned in the maximal tolerable dorsiflexion. As soon as the participant reached their maximum ROM, this position was held for two minutes. They were instructed to re-adjust the position of the ankle (using the remote control of the dynamometer), if the passive tension decreased and encouraged to provide a constant stretch of maximum tolerable intensity. For the V and the STV conditions, a vibration dumbbell “TremoFlex” (TremoTec GmbH, Oberstdorf, Germany) was positioned at the upper part of the gastrocnemius muscle right above the ProbeFix. Frequency of vibration was set to 30 Hz, to allow a better comparison to previous studies as it is the most commonly used vibration frequency in vibration literature (Jochum et al. 2025a) with respective peak-to-peak amplitude of 4 mm and calculated acceleration of 142 m/s^2^ (14.5 g) (Cochrane 2011b).

### Data analysis and statistical processing

Data were processed using Microsoft Excel (Version 16.92, Microsoft Corporation, Redmond, WA, USA). The maximum ROM (ROMmax) of the three trials of pre- as well as post-testing was used for statistical analysis. Stiffness values from the three ultrasound snapshots were averaged for pre-and post-test, respectively for resting position and ROMmax. Passive torque–angle curves were visually inspected and, if appropriate, two valid cycles were averaged to one-degree-steps. If measurements were considered as not valid (e.g. stiffness measurements saturated and presented measurement gaps; active contraction occurred during passive torque assessment), these data were excluded from further analysis. Passive resting torque (PRT) was extracted at resting position (20° ankle plantarflexion), and averaged over all values when the device did not move (0 m/s) from valid cycles. Similarly, passive peak torque (PPT) was extracted at ROMmax (device stagnant and rated as valid). Absolute passive torque was further prepared for data analysis in relation to the relative ROM of each participant in 10% steps, whereby 100% represented the maximum ROM from the pre-test. Accordingly, the same ROM-relation was applied to the post-test to compare the respective angle concerning the related passive torque. Therefore, the passive torque measured at the maximum angle of the pre-test (PT-100) was used to compare it to the passive torque at the same angle after the intervention. For presentation purposes, the corresponding relative passive torque (100% representing the maximum torque from the pre-test) was outlined graphically with MATLAB (R2023a, The MathWorks Inc., Natick, MA, USA).

Data were evaluated using JASP (Version 0.19.1, JASP Team 2024, Amsterdam, Netherlands). Normal distribution of main outcome parameters was tested using the Shapiro–Wilk test, but repeated measures ANOVA with high sample sizes (> 30 observations) are robust against deviations from normal distribution (Wilcox 2011). Reliability between trials per day and between days were determined by calculating two-way intraclass correlation coefficient (ICCs) for agreement (Ko et al. 2020). Additionally, to track day-to-day variance in baseline values, a one-way analysis of variance (ANOVA) was applied. Whether, or not, superimposing vibration to stretching provides additional benefits compared to the other groups was investigated by applying a two-way ANOVA with repeated measurements. Post hoc testing for intergroup differences in pre- to post-changes was performed using the Scheffé test, while for intragroup differences the Bonferroni correction was applied. Effect sizes were interpreted as follows: small: *η*_*p*_^2^ < 0.06, moderate: *η*_*p*_^2^ = 0.06–0.14, large: *η*_*p*_^2^ ≥ 0.14. Cohen’s d was calculated for individual group comparisons and was classified as: trivial: *d* = 0–0.2, small: *d* = 0.2–0.5, moderate: *d* = 0.5–0.8, large: *d* ≥ 0.8 (Cohen 1992). The level of significance was set to *α* = 0.05.

## Results

The absence of three participants at their final investigations as well as inspected non-validity of torque (PRT: *n* = 14, PT-100: *n* = 7, PPT: *n* = 4) or stiffness (Rest: *n* = 16, ROMmax: *n* = 41) assessments led to exclusion of several data points and are therefore reported with different sample sizes in Table 1. The Shapiro–Wilk test indicated normal distribution for ROM (*p* = 0.147–0.327), but not for stiffness and torque parameters (*p* < 0.001–0.119). ICCs for interday reliability indicated sufficient to high reliability over the different times showing ICCs ranging between 0.77–0.96 (see Supplemental Table 1). Pre-test values were not significantly different between the interventions (*p* = 0.226–0.503) except for passive resistive torque in the resting position (*p* = 0.049).

**Table 1:** Results of repeated measures ANOVA for range of motion (ROM), stiffness at rest (20° plantarflexion) as well as peak ROMmax, and passive torque at rest (PRT), at ROMmax from Pre-Test (PT-100) as well as at peak ROMmax (PPT) before (Pre) and after (Post) the interventions stretching + vibration (STV), stretching alone (ST), vibration alone (V) and control group (CG) with overall (Time) and interaction (Time X Int) effect as well as post-hoc intragroup changes with Bonferroni adjustment (*p* value) if “Time X Int” was significant

		*n*	Pre	Post	*p* value	Time	Time X Int
ROM (°)	STV	30	21.0 ± 12.3	25.3 ± 12.6^a^	< 0.001	*F*_113, 1_= 185.616	*F*_113, 3_ = 16.158
DF Max	ST	28	23.2 ± 12.9	26.7 ± 13.3^a^	< 0.001	*p*< 0.001	*p* < 0.001
	V	30	22.6 ± 11.7	26.1 ± 12.6^a^	< 0.001	*η*^2^= 0.622	*η*^2^= 0.300
	CG	29	22.6 ± 11.6	23.0 ± 11.6	0.348		
PRT (Nm)	STV	26	3.1 ± 1.9	2.7 ± 2.0	0.007	*F*_100, 1_= 6.315	*F*_100, 3_= 3.418
Rest	ST	25	2.6 ± 1.5	2.6 ± 1.7	0.871	*p* = 0.014	*p* = 0.020
(20° Plantarflexion)	V	26	2.9 ± 2.0	2.4 ± 1.6	0.004	*η*^2^= 0.059	*η*^2^=0.093
	CG	26	2.6 ± 1.7	2.7 ± 1.8	0.613		
PT-100 (Nm)	STV	29	46.0 ± 23.9	41.8 ± 22.9^a,b^	< 0.001	*F*_106, 1_= 79.417	*F*_106_, 3 = 23.993
ROMmax (PreTest)	ST	26	48.9 ± 27.9	45.9 ± 26.6	< 0.001	*p* < 0.001	*p* = 0.002
	V	30	46.0 ± 25.5	44.5 ± 24.6	0.007	*η*^2^= 0.428	*η*^2^= 0.132
	CG	25	47.4 ± 26.3	45.8 ± 26.1	0.012		
PPT (Nm)	STV	30	44.7 ± 23.9	52.9 ± 25.7^a^	< 0.001	*F*_109, 1_= 69.674	*F*_109, 3_ = 8.058
ROMmax	ST	27	47.7 ± 27.5	55.1 ± 31.6^a^	< 0.001	*p* < 0.001	*p* < 0.001
	V	30	45.4 ± 24.6	54.3 ± 29.4	< 0.001	*η*^2^= 0.390	*η*^2^= 0.182
	CG	26	45.8 ± 25.9	45.7 ± 26.6	0.897		
Stiffness (kPa)	STV	26	22.3 ± 5.3	21.4 ± 6.7		*F*_97, 1_ = 8.070	*F*_97, 3_ = 1.174
Rest	ST	25	24.2 ± 7.0	21.8 ± 5.6		*p*= 0.005	*p* = 0.324
(20° Plantarflexion)	V	26	24.7 ± 6.1	23.5 ± 5.9		*η*^2^= 0.077	*η*^2^ = 0.035
	CG	24	25.1 ± 5.4	24.9 ± 4.8			
Stiffness (kPa)	STV	20	218.2 ± 111.1	247.1 ± 116.0		*F*_72, 1_= 5.502	*F*_72, 3_ = 1.454
ROMmax	ST	18	242.8 ± 98.1	258.5 ± 110.4		*p* = 0.022	*p* = 0.234
	V	19	245.0 ± 97.6	264.5 ± 115.0		*η*^2^= 0.071	*η*^2^ = 0.057
	CG	19	233.5 ± 79.2	227.4 ± 79.5			

^a^Significant group difference against the control group

^b^Significant group difference against vibration alone

### Range of motion

**Fig. 2:**
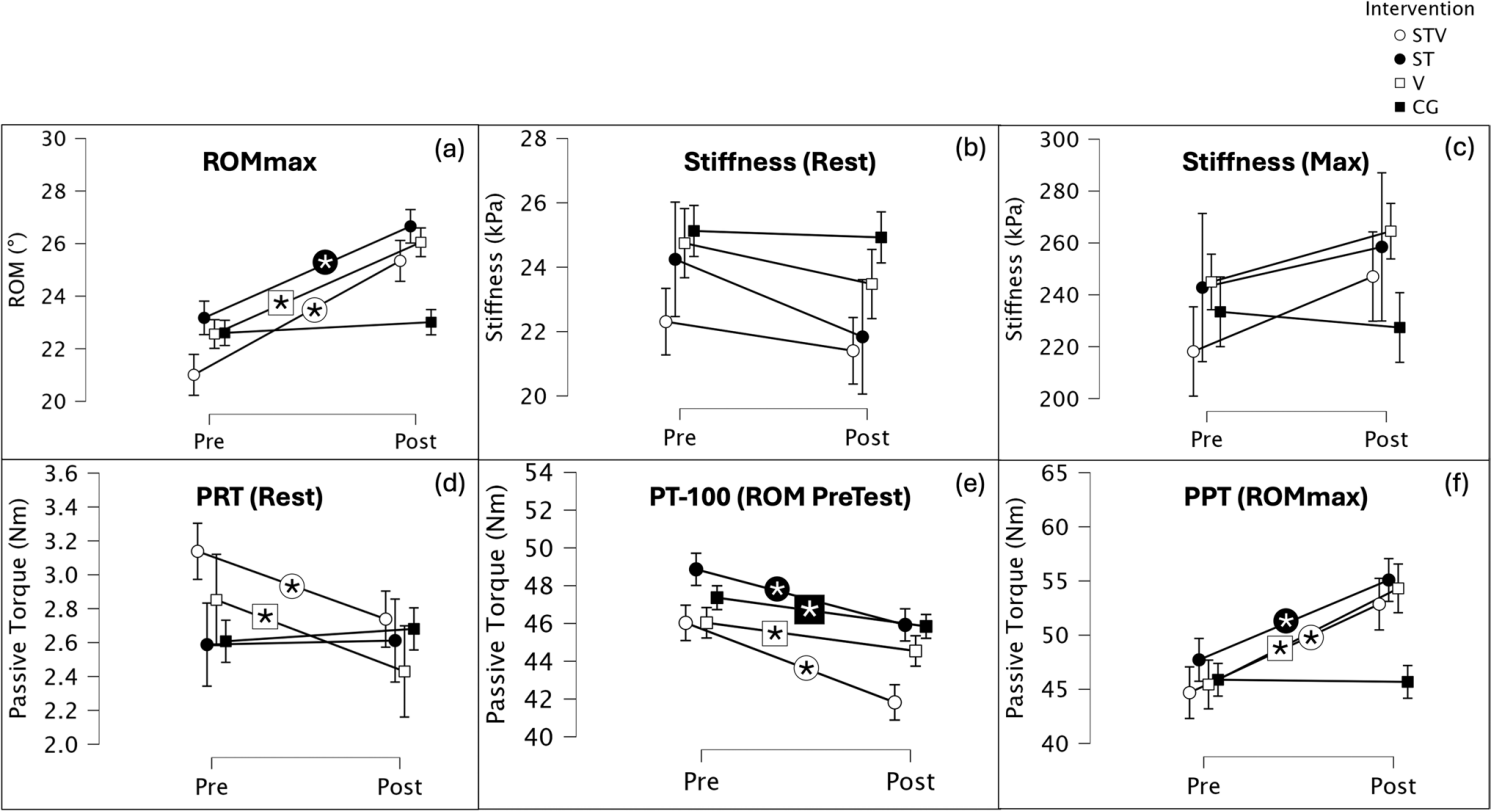
Group values (mean ± SD) for **a** range of motion (ROM), **b** stiffness at rest, **c** stiffness at ROMmax and **d** passive torque at rest (PRT), **e** passive torque at ROMmax from Pre-Test (PT-100) and **f** passive peak torque at ROMmax (PPT) before (Pre) and after (Post) the interventions stretching + vibration (STV), stretching alone (ST), vibration alone (V) and control group (CG) with stars (*) indicating significant intragroup changes with Bonferroni adjustment from Pre to Post in the respective group

There was a significant large magnitude increase (*F*_113,1_ = 185.6, *p* < 0.001, *η*^2^ = 0.622) in ROM over all groups (Table 1), while the interaction effect (*F*_113,3_ = 16.2, *p* < 0.001, *η*^2^ = 0.300) indicated significant group differences (Fig. 2). All interventions increased significantly (*p* < 0.001) and showed higher increases of large magnitude compared to the control condition (STV: *p* < 0.001, *d* = 1.560, ST: *p* < 0.001, *d* = 1.184 and V: *p* < 0.001, *d* = 1.188), while no significant difference could be determined between the interventions (*p* = 0.56 – 1.0).

### Muscle stiffness

Gastrocnemius medialis muscle stiffness in the resting position (20° plantarflexion) showed a significant decrease in all interventions (*F*_97,1_ = 8.1, *p* = 0.005, *η*^2^ = 0.077). The interaction effect did not reach the level of significance (*F*_97,3_ = 1.2, *p* = 0.324, *η*^2^ = 0.035) (Table 1).

Muscle stiffness at ROMmax showed an increase over all conditions (*F*_72, 1_ = 5.5, *p* = 0.022, *η*^2^ = 0.071), while the interaction effect revealed no significant difference between groups (*F*_72, 1_ = 1.5, *p* = 0.234, *η*^2^ = 0.057).

### Passive resistive torque

The passive resistive torque in the resting position (20° plantarflexion) revealed a significant, small main effect (*F*_100,1_ = 6.3, *p* = 0.014, *η*^2^ = 0.059) with a significant interaction effect (*F*_100,3_ = 3.4, *p* = 0.020, *η*^2^ = 0.093) (Table 1). STV (*p* = 0.007) and V (*p* = 0.004) showed significant decreases, but the comparison with the other groups did not reach significance (*p* = 0.152–1.00).

Comparing the passive torque at the ROMmax from the Pre-Test (PT-100) with the equivalent joint position in the Post-Test (Table 1) showed a large significant decrease over all groups (*F*_106,1_ = 79.4, *p* < 0.001, *η*^2^ = 0.428) as well as a moderate interaction effect (*F*_106,3_ = 24.0, *p* = 0.002, *η*^2^ = 0.132). All conditions decreased significantly (STV: *p* < 0.001, ST: *p* < 0.001, V: *p* = 0.007, CG: *p* = 0.012). Post-hoc testing indicated significant higher decreases of STV compared with V (*p* = 0.009) and CG (*p* = 0.015).

Passive peak torque at ROMmax showed a significant main effect (*F*_109,1_ = 69.7, *p* < 0.001, *η*^2^ = 0.390) with also significant interaction between interventions (*F*_109,3_ = 8.1, *p* < 0.001, *η*^2^ = 0.182). STV, ST, and V showed significant increases (*p* < 0.001) which were largely different to the steady level of the CG (CG vs. STV: *p* < 0.001, *d* = 1.115; CG vs. ST: 0.004, *d* = 1.212; CS vs. V: *p* < 0.001, *d* = 1.260).

**Fig. 3:**
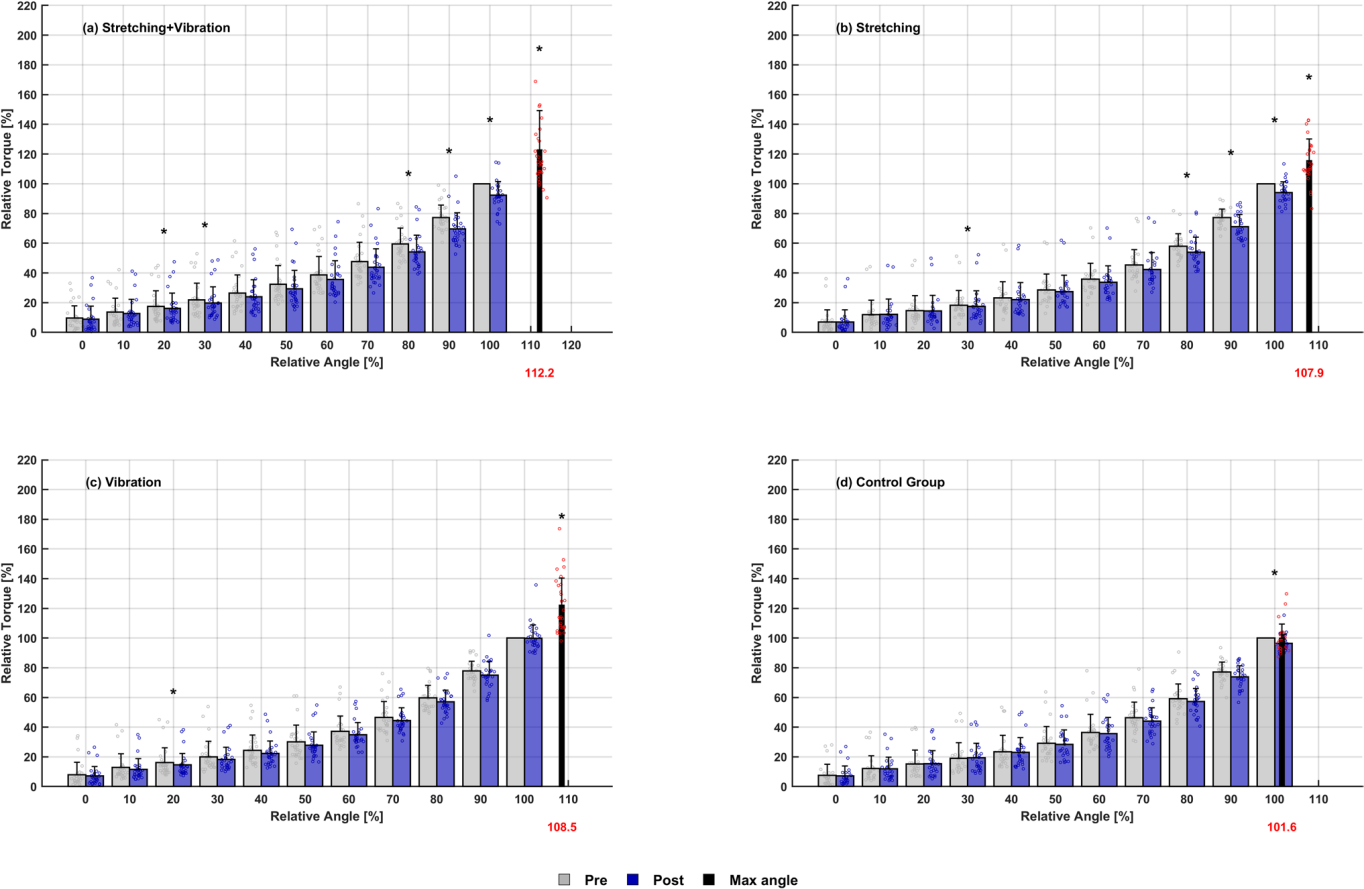
Relative passive torque (in relation to the maximum torque from the pre-test as 100%) in relation to the maximum range of motion (relative angle) from the pre-test (100%) in 10% stages from before (Pre: grey) to after (Post: blue) the interventions with significant pre-post differences marked (*) for **a** stretching + vibration (STV), **b** stretching alone (ST), **c** vibration alone (V) and **d** control group (CG)

The passive torque–angle curve over the whole ROM, which was compared in relation to the maximum ROM from the pre-test (100%) (Table 2), showed significant interaction effects at 20–30% ROM (*p* = 0.005–0.042) and from 80–100% ROM (*p* = 0.005–0.008) with significant decreases for STV (*p* < 0.001) and ST (*p* < 0.001–0.318). Further, STV showed significant decreases in all relative ROMs below 100% (*p* < 0.001), while V alone only in lower ROM 20% (*p* = 0.011) (Fig. 3).

**Table 2:** Absolute passive torque (PT) values (Nm) in relation to the maximum range of motion (ROM) from the pre-test (100%) in 10% stages before (Pre) and after (Post) the interventions stretching + vibration (STV), stretching alone (ST), vibration alone (V) and control group (CG) with main and interaction effect from repeated measures ANOVA and the intragroup changes with Bonferroni adjustment for each group in the respective row (if interaction was significant)

	Pre	Post	*p* value	Pre	Post	*p* value	Pre	Post	*p* value	Pre	Post	*p* value
Main	PT 0%	F_99,1_ = 17.2	0.005	PT 10%	F_105,1_ = 9.6	0.002	PT 20%	F_105,1_ = 11.9	< 0.001	PT 30%	F_105,1_ = 10.5	0.002
Interaction		F_99,3_ = 2.5	0.187		F_105,3_ = 2.1	0.110		F_105,3_ = 2.8	0.042		F_105,3_ = 4.6	0.005
STV	3.3 ± 2.1	2.9 ± 2.2		4.8 ± 2.5	4.4 ± 2.3		6.2 ± 2.6	5.7 ± 2.4	< 0.001	7.9 ± 3.1	7.2 ± 2.9	< 0.001
ST	2.4 ± 1.5	2.3 ± 1.6		4.2 ± 2.0	4.2 ± 2.1		5.6 ± 2.5	5.5 ± 2.5	0.318	7.3 ± 2.9	6.9 ± 2.8	0.038
V	2.8 ± 2.0	2.5 ± 1.5		4.6 ± 2.1	4.3 ± 1.9		5.9 ± 2.3	5.5 ± 2.2	0.011	7.5 ± 2.7	7.1 ± 2.8	0.059
CG	2.5 ± 1.9	2.5 ± 1.7		4.3 ± 2.2	4.3 ± 2.3		5.6 ± 2.7	5.7 ± 2.8	0.814	7.2 ± 3.2	7.5 ± 3.5	0.190
Main	PT 40%	F_105,1_ = 17.7	< 0.001	PT 50%	F_105,1_ = 17.2	< 0.001	PT 60%	F_105,1_ = 17.1	< 0.001	PT 70%	F_105,1_ = 13.9	< 0.001
Interaction		*F*_105,3_ = 1.9	0.136		*F*_105,3_ = 2.5	0.217		*F*_105,3_ = 1.2	0.302		*F*_105,3_ = 1.2	0.322
STV	9.9 ± 3.9	8.9 ± 3.7		12.4 ± 4.7	11.2 ± 4.8		15.2 ± 6.3	13.9 ± 6.3		19.1 ± 8.4	17.6 ± 8.5	
ST	9.5 ± 4.0	9.0 ± 3.8		12.2 ± 5.2	11.6 ± 4.7		15.7 ± 7.4	14.6 ± 6.4		20.4 ± 10.1	18.8 ± 9.1	
V	9.3 ± 3.2	8.8 ± 3.3		11.6 ± 3.9	11.1 ± 4.3		14.8 ± 5.6	14.2 ± 5.9		18.8 ± 7.1	18.6 ± 8.8	
CG	9.2 ± 4.2	9.0 ± 3.9		11.7 ± 5.4	11.4 ± 5.0		14.8 ± 6.8	14.4 ± 6.5		19.6 ± 10.4	18.5 ± 9.3	
Main	PT 80%	F_105,1_ = 33.7	< 0.001	PT 90%	F_105,1_ = 45.3	< 0.001	PT 100%	F_105,1_ = 30.2	< 0.001	PT Max	F_105,1_ = 68.4	< 0.001
Interaction		F_105,3_ = 4.5	0.005		F_105,3_ = 4.6	0.005		F_105,3_ = 4.2	0.008		*F*_105,3_ = 9.4	< 0.001
STV	24.4 ± 11.4	22.0 ± 10.0	< 0.001	32.3 ± 15.4	28.9 ± 13.9	< 0.001	42.8 ± 21.5	39.4 ± 20.6	< 0.001	42.8 ± 21.5	51.0 ± 23.8	< 0.001
ST	27.1 ± 14.8	25.1 ± 13.9	< 0.001	37.1 ± 21.6	34.4 ± 21.3	< 0.001	48.7 ± 28.2	46.2 ± 28.0	< 0.001	48.7 ± 28.2	55.7 ± 32.8	< 0.001
V	24.9 ± 11.1	24.3 ± 12.3	0.170	33.3 ± 16.4	32.4 ± 17.8	0.151	43.6 ± 22.7	43.4 ± 23.0	0.778	43.6 ± 22.7	52.9 ± 28.7	< 0.001
CG	25.3 ± 13.7	24.9 ± 14.7	0.402	33.7 ± 18.7	32.6 ± 19.3	0.094	44.3 ± 25.1	42.9 ± 25.7	0.044	44.3 ± 25.1	43.7 ± 25.2	0.698

## Discussion

Contrary to the initially stated hypothesis, superimposed vibration caused no additional significant benefits on flexibility. Furthermore, vibration alone demonstrated effects comparable to those of stretching, consistent with the results of recent research (Warneke et al. 2024a; Jochum et al. 2025a). Beyond the evaluation of the main outcome ROM, differences between interventions were found in proposed underlying mechanisms, where vibration decreased passive resistive torque in rest, while no group difference for resting muscle stiffness was found. When examining the passive torque in comparison to the pre-test measurement at the respective relative ROM steps (%), the combination of vibration and stretching showed pronounced effects on passive torque decreases over the whole ROM.

### Underlying mechanisms of vibration

Although no superior ROM effects were detected, our assumption that vibration resulted in improved ROM and related parameter adaptation stems from recent scientific literature that has extensively discussed underlying physiological mechanisms. More specifically, vibration was suggested to alter neural (Cochrane 2011a), circulatory and thermoregulatory systems (Fowler et al. 2019) as well as mechano-chemical signal conversion (Lewis et al. 2017) and pain sensation. Considering the muscle tuning theory (Nigg 1997; Cochrane 2011a), several authors suggested that the musculoskeletal structures must quickly modulate muscle stiffness to cope with the vibratory waves (Cardinale and Bosco 2003). Hereby, vibration elicits a reflexive muscle contraction—called the tonic vibration reflex (TVR) (Issurin et al. 1994)—over type-Ia afferent fibres (Lance et al. 1973) to dampen the vibratory waves. Added contractions in high muscle lengths (e.g. electromyostimulation) were already shown effective in further enhancing the end ROM (Mizuno 2023), but are unclear considering muscle stiffness. According to our results, neither vibration nor stretching of two minutes seemed sufficient to affect resting muscle stiffness, while the joint resistance measured with the passive resistive torque was reduced only with vibration.

Mechanical vibration also enhances blood flow and temperature (Fowler et al. 2019) and thus acting on thermo-sensory transient receptor potential ion channels (Vay et al. 2010, 2012). The main passive resistive component of muscle tissue is given by intramuscular collagen type I fibres and their fibre architecture (Taylor et al. 1990; Fratzl et al. 1998), which slightly extends (Warren et al. 1976) and decreases Young’s modulus (Gevorkian et al. 2009) with increasing temperature. However, as additional vibration did not affect resting muscle stiffness, but was shown to reduce apparent joint stiffness, it is possible that other structures than the muscle were affected, e.g. tendons or muscle–tendon junctions (Konrad et al. 2019).

Furthermore, mechanical-gated Piezo-Channels for stretch, pain as well as proprioception sensing (Savadipour et al. 2023) are activated by vibration (Lewis et al. 2017). Some authors proposed analgesic effects of vibration (Lundeberg et al. 1984; Pantaleo et al. 1986), especially local vibration with high frequencies acting on the spinal gate control (Casale and Hansson 2022). This is in line with our results as also vibration alone led to significant increases in PPT.

Concerning the vibration parameters, a frequency of 26 Hz was shown to elicit the greatest EMG response for a given muscle (Cochrane et al. 2008). Derived from the muscle activation, it was suggested that muscle tone decreases with low frequencies (8–15 Hz) and increases with high frequencies (20–35 Hz) (Rittweger 2020)—though this relation was never demonstrated with direct measurements of muscle stiffness (Cardinale and Bosco 2003; Cronin et al. 2004). In contrast, though we used a potentially high frequency with 30 Hz, joint resistance was decreased by vibration as well as stretching with superimposed vibration. Interestingly, Di Giminiani et al. (2010) and Masud et al. (2022) highlighted that the optimal frequency is individual and even muscle-dependent, respectively. This might be related to the transmission of the vibratory waves (Friesenbichler et al. 2014), which are also strongly dependent on vibration frequency (Cidem et al. 2017) and change in relation to posture (Fratini et al. 2009), with muscle length determining the TVR response (Nordin and Hagbarth 1996). Wang et al. (2007) showed that the so-called resonance frequency of the patellar tendon—measured by its displacement with an accelerometer in response to vibration frequencies from 15 to 60 Hz—increased with knee flexion, thus with tension on the tendon itself. Therefore, though hypothesized to be effective in stretched position (Cardinale and Lim 2003; Ritzmann et al. 2013), altered muscle activations might call for adapted vibration protocols to elevate effects.

Taken together, these mechanisms influence ROM on different levels, distinguishing between structural and neuronal parameters which can be reported separately and related to a more *mechanical* and a *sensory* theory.

### Mechanical theory and vibration effects on joint resistance

Following the *mechanical theory,* authors attributed ROM increases to a diminished passive joint stiffness at rest (i.e. joint passive torque change per angle change) or changes in mechanical properties (i.e. alterations of tissue stiffness) (Freitas et al. 2018). Our results indicate that ROM increases are mediated differently between interventions, with stretching affecting stiffness, while vibration affected the passive torque. Though additional vibration did not further enhance ROM of stretching, it was shown to decrease apparent joint resistance over the whole ROM. This might result from the combination of interventions, as vibration alone affected just the lower segment up to 40% ROM, while stretching affected the segment above 60% ROM.

It is well known that stretching is a sufficient moderator for muscle and connective tissue stiffness. Konrad et al. (2017b) and Konrad et al. (2019) found reductions of muscle stiffness (up to 25%) and muscle–tendon stiffness (− 10.5 to − 13.7%) with up to five minutes of (either static, dynamic, and ballistic). Additionally, Warneke et al. (2024b) showed that not only muscle, but also the surrounding connective tissue acutely reduced their stiffness when imposing stretching for 1 × 5 min. Accordingly, the presented results are in line with previous reports: muscle stiffness at rest was decreased only with stretching (Takeuchi et al. 2023).

However, a recent meta-analysis (Jochum et al. 2025a) suggested that vibration could provide additional benefits when moderating muscle stiffness (Effect size (ES) = − 0.89, *p* = 0.006), which stems from studies that opposed superimposed vibration (vibration + additional exercises) against a passive control group. Interestingly, the comparison against the same exercise without vibration (sham) showed no significant effect (ES = − 0.19, *p* = 0.076). Since no additional benefits of vibration on acute muscle stiffness adaptations were observed, but as passive torque seems to be affected, the relevance of vibration for other tissue changes (stiffness) needs further investigation to point out benefits, if any exist.

### Sensory theory and vibration effects on pain threshold

According to the *sensory theory* of ROM increases, interventions like stretching or vibration might reduce the sensibility for stretching pain (i.e. increased pain threshold) with negligible structural adaptations (Freitas et al. 2018; Weppler and Magnusson 2010). As the pain threshold limits stretching movements (Alter 2004), the passive peak torque (PPT) measured in the end ROM determines the highest stretch torque participants can tolerate (Folpp et al. 2006). In contrast to the belief that vibration provides additional benefits, no statistically significant differences in PPT improvements were observed between the interventions, with all conditions leading to comparable increases. Therefore, these findings question postulated altered pain sensation (Lundeberg et al. 1984; Pantaleo et al. 1986), via the spinal gate control (Casale and Hansson 2022) induced by high vibration frequencies, as these theories stem from passively controlled research. Vibration at certain frequencies reflexively induces muscle contraction, which can lead to tissue warming (Fuller et al. 2013) and subsequent alterations in tissue properties, potentially influencing the observed effects (Jochum et al. 2025a). Interestingly, there was just one similar study conducted by Rodrigues et al. (Rodrigues et al. 2017), who found similar increases for ROM and maximum PPT. Therefore, it seems that potential vibration effects, at least on acute ROM and PPT remain subjective (Issurin 2005). However, due to the very limited number of articles reporting reasonable study designs, further research is warranted to address mechanisms.

### Acute warm-up effects

In the literature, several authors explored the acute effects of specific training interventions on ROM and underlying mechanisms such as PPT or stiffness by opposing intervention effects to passive control conditions (Wilke et al. 2020; Behm et al. 2023). While this procedure is very common, the validity of the made interpretations must be questioned as the influence of generally enhanced activity and resulting warm-up effects cannot be ruled out (Warneke et al. 2024a). Since improved blood circulation and heat generation are linked to enhanced flexibility (Issurin 2005), additional heating was shown to further increase flexibility effects of stretching interventions by a meta-analysis (Nakano et al. 2012). Therefore, to investigate whether there are specific effects of adding vibration to stretching, active comparisons (ST and V) were included in addition to a passive control in this study. Although given the discussion on specific vibration effects from literature (e.g. increased the blood flow via mechanical vibration stimuli (Fuller et al. 2013) which results in a subsequent heat generation (Oliveri et al. 1989)), no such effects were obtained in this study: All interventions improved ROM to a similar magnitude and no significant differences between STV, V and ST were observed, while the passive control condition showed no statistically and practically significant ROM increments from pre- to post.

### Limitation

The habituation effect concerning the ROM and passive torque assessment can be seen over the course of the measurement. To counteract potential warm-up effects within one testing session, we provided a specific warm-up and measured the highest ROM from three valid measurements, as the values increased consistently from first to second but less with the third measurement. However, it cannot be ruled out that further increases could be measured when continuing the warm-up stretches. Therefore, a standardized testing protocol should be evaluated to ensure consistency and reliability in PPT investigations and ROM assessments using the dynamometer device. Although participants were allowed to decide individually when to terminate the testing, the protocol demonstrated good intraday and interday reliability, mitigating potential concerns about variability. Additionally, it should be noted that the control condition, labelled as 'no intervention' with passive rest, involved six ROM assessments and six passive torque assessments, which could arguably be interpreted as a form of 'dynamic stretching.' This could have contributed to the significant improvements observed in passive torque (PT-100), further highlighting the need for a strictly standardized approach in future studies. Noteworthy, especially for PT-100 and PPT, we had to exclude some values (with a focus on the CG) due to diverse reasons (see Results section for measurement errors). In theory, as evaluated via ANOVAs, the fact of different sample sizes could have affected the statistical analysis, as the variance depends on the sample size. However, this deviation in sample size is limited to just few individual data points. As currently no standardized measurement guidelines exist, this might call for investigations concerning the consistency of repeated ROM and torque measurements. In this manner, one must consider that participants with an alleged low ROM might show higher (relative) increases in comparison to participants with already high flexibility. As “more flexible” persons might potentially have an increased sensibility for specific stretching pain, this might have led to the broad distribution of passive torque and stiffness values. This may also be influenced by the testing and intervention position with 110° hip flexion, potentially altering ROM (Andrade et al. 2016) as well as stretching of the sciatic nerve (Andrade et al. 2018). As we conducted a repeated-measures design, the intra-individual comparisons required a standardized assessment and intervention position, which did not depend on the involvement of the sciatic nerve. Nevertheless, this should be considered and even tested in future investigations. Still a limitation, but this might explain the missing normal distribution of these parameters, calling for a more decent selection or segmentations of participants in future studies. Though ANOVAs with high sample sizes are very robust against deviations from normal distributions (Wilcox 2011), we compared our results with the non-parametric Kruskal–Wallis test and found similar results while some became also significant (PT-0: *p* = 0.013; PT-40: *p* = 0.004; PT-50: *p* = 0.007; PT-60: *p* = 0.020). In this manner, though the statistical analysis with variance analysis for higher statistical power provided significance for several interaction effects, the post hoc analysis missed several times the statistical significance for group differences. Of course, this questions the significance of our findings, but we want to refer also to the clinical relevance (Willigenburg and Poolman 2023; Page 2014): especially since stretching in combination with vibration produced a five percent higher increase in ROM in comparison to the separated comparison groups.

## Conclusion

Vibration is not superior to stretching but might be used as an alternative to stretching to increase ROM immediately and is potentially interesting as a warm-up in the resting condition. Though the superimposition of vibration to stretching did not further increase ROM, the combination of both interventions presented the most prominent influence on passive torque. This might be interesting for practitioners, who struggle with progress in flexibility performance using conventional stretching exercises. As vibration affects joint resistance rather than muscle stiffness, other structures might be affected, calling for further research of this topic. As vibration was proposed to induce analgesic effects, this might facilitate the conduction of exercises and improves compliance as well as adherence of stretching habits. Therefore, vibration could provide a useful and interesting alternative for flexibility.

## Supplementary Information

Below is the link to the electronic supplementary material.

Supplementary file1 (DOCX 28 KB)

## Data Availability

All data generated or analysed during this study are included in this published article and its supplementary information files. Datasets are available from the corresponding author upon reasonable request.

## Abbreviations

ROM: Range of motion
STV: Stretching + vibration
V: Vibration
ST: Stretching
CG: Passive control
PNF: Proprioceptive neuromuscular facilitation
TVR: Tonic vibration reflex
WBV: Whole-body vibration
PRT: Passive resting torque
PPT: Passive peak torque at end ROM
PT-100: Passive torque measured at the maximum angle of the pre-test
SWE: Shear-wave elastography
ROMmax: Maximum range of motion
ICC: Intraclass correlation coefficient
ANOVA: Analysis of variance

## References

[CR1] Alter MJ (2004) Science of flexibility. Human Kinetics, Champaign

[CR3] Andrade RJ, Lacourpaille L, Freitas SR, McNair PJ, Nordez A (2016) Effects of hip and head position on ankle range of motion, ankle passive torque, and passive gastrocnemius tension. Scand J Med Sci Sports 26(1):41–47. 10.1111/sms.12406 25676048 10.1111/sms.12406

[CR2] Andrade RJ, Freitas SR, Hug F, Le Sant G, Lacourpaille L, Gross R, McNair P, Nordez A (2018) The potential role of sciatic nerve stiffness in the limitation of maximal ankle range of motion. Sci Rep 8(1):14532. 10.1038/s41598-018-32873-6 30266928 10.1038/s41598-018-32873-6 PMC6162234

[CR4] Behm DG, Alizadeh S, Daneshjoo A, Anvar SH, Graham A, Zahiri A, Goudini R, Edwards C, Culleton R, Scharf C, Konrad A (2023) Acute effects of various stretching techniques on range of motion: a systematic review with meta-analysis. Sports Med Open 9(1):107. 10.1186/s40798-023-00652-x 37962709 10.1186/s40798-023-00652-x PMC10645614

[CR5] Bradley PS, Portas MD (2007) The relationship between preseason range of motion and muscle strain injury in elite soccer players. J Strength Cond Res 21(4):1155–1159. 10.1519/r-20416.1 18076233 10.1519/R-20416.1

[CR6] Cardinale M, Bosco C (2003) The use of vibration as an exercise intervention. Exerc Sport Sci Rev 31(1):3–7. 10.1097/00003677-200301000-00002 12562163 10.1097/00003677-200301000-00002

[CR7] Cardinale M, Lim J (2003) Electromyography activity of vastus lateralis muscle during whole-body vibrations of different frequencies. J Strength Cond Res 17(3):621–624. 10.1519/1533-4287(2003)017%3c0621:eaovlm%3e2.0.co;2 12930196 10.1519/1533-4287(2003)017<0621:eaovlm>2.0.co;2

[CR8] Casale R, Hansson P (2022) The analgesic effect of localized vibration: a systematic review. Part 1: the neurophysiological basis. Eur J Phys Rehabil Med 58(2):306–315. 10.23736/s1973-9087.22.07415-9 35102735 10.23736/S1973-9087.22.07415-9 PMC9980599

[CR9] Cidem M, Karacan I, Cakar HI, Cidem M, Sebik O, Yilmaz G, Turker KS, Karamehmetoglu SS (2017) Vibration parameters affecting vibration-induced reflex muscle activity. Somatosens Mot Res 34(1):47–51. 10.1080/08990220.2017.1281115 28152665 10.1080/08990220.2017.1281115

[CR10] Cochrane DJ (2011a) The potential neural mechanisms of acute indirect vibration. J Sports Sci Med 10(1):19–30 24149291 PMC3737901

[CR11] Cochrane DJ (2011b) Vibration exercise: the potential benefits. Int J Sports Med 32(2):75–99. 10.1055/s-0030-1268010 21165804 10.1055/s-0030-1268010

[CR12] Cochrane DJ, Stannard SR, Walmsely A, Firth EC (2008) The acute effect of vibration exercise on concentric muscular characteristics. J Sci Med Sport 11(6):527–534. 10.1016/j.jsams.2007.04.006 17714990 10.1016/j.jsams.2007.04.006

[CR13] Cohen J (1992) Statistical power analysis. Curr Dir Psychol Sci 1(3):98–101

[CR14] Cronin JB, Oliver M, McNair PJ (2004) Muscle stiffness and injury effects of whole body vibration. Phys Ther Sport 5(2):68–74

[CR15] Di Giminiani R, Manno R, Scrimaglio R, Sementilli G, Tihanyi J (2010) Effects of individualized whole-body vibration on muscle flexibility and mechanical power. J Sports Med Phys Fit 50(2):139–151 20585287

[CR16] Folpp H, Deall S, Harvey LA, Gwinn T (2006) Can apparent increases in muscle extensibility with regular stretch be explained by changes in tolerance to stretch? Aust J Physiother 52(1):45–50. 10.1016/s0004-9514(06)70061-7 16515422 10.1016/s0004-9514(06)70061-7

[CR17] Fowler BD, Palombo KTM, Feland JB, Blotter JD (2019) Effects of whole-body vibration on flexibility and stiffness: a literature review. Int J Exerc Sci 12(3):735–747 31156749 10.70252/TJVC4921 PMC6533098

[CR18] Fratini A, La Gatta A, Bifulco P, Romano M, Cesarelli M (2009) Muscle motion and EMG activity in vibration treatment. Med Eng Phys 31(9):1166–1172. 10.1016/j.medengphy.2009.07.014 19671494 10.1016/j.medengphy.2009.07.014

[CR19] Fratzl P, Misof K, Zizak I, Rapp G, Amenitsch H, Bernstorff S (1998) Fibrillar structure and mechanical properties of collagen. J Struct Biol 122(1–2):119–122. 10.1006/jsbi.1998.3966 9724612 10.1006/jsbi.1998.3966

[CR20] Freitas SR, Mendes B, Le Sant G, Andrade RJ, Nordez A, Milanovic Z (2018) Can chronic stretching change the muscle-tendon mechanical properties? a review. Scand J Med Sci Sports 28(3):794–806. 10.1111/sms.12957 28801950 10.1111/sms.12957

[CR21] Friesenbichler B, Lienhard K, Vienneau J, Nigg BM (2014) Vibration transmission to lower extremity soft tissues during whole-body vibration. J Biomech 47(12):2858–2862. 10.1016/j.jbiomech.2014.07.028 25128392 10.1016/j.jbiomech.2014.07.028

[CR22] Fuller JT, Thomson RL, Howe PR, Buckley JD (2013) Effect of vibration on muscle perfusion: a systematic review. Clin Physiol Funct Imaging 33(1):1–10. 10.1111/j.1475-097X.2012.01161.x 23216759 10.1111/j.1475-097X.2012.01161.x

[CR23] Garber CE, Blissmer B, Deschenes MR, Franklin BA, Lamonte MJ, Lee IM, Nieman DC, Swain DP (2011) American College of Sports Medicine position stand. Quantity and quality of exercise for developing and maintaining cardiorespiratory, musculoskeletal, and neuromotor fitness in apparently healthy adults: guidance for prescribing exercise. Med Sci Sports Exerc 43(7):1334–1359. 10.1249/MSS.0b013e318213fefb 21694556 10.1249/MSS.0b013e318213fefb

[CR24] Gevorkian SG, Allahverdyan AE, Gevorgyan DS, Simonian AL (2009) Thermal (in)stability of type I collagen fibrils. Phys Rev Lett 102(4):048101. 10.1103/PhysRevLett.102.048101 19257477 10.1103/PhysRevLett.102.048101

[CR25] Hagbarth KE, Eklund G (1966) Tonic vibration reflexes (TVR) in spasticity. Brain Res 2(2):201–203. 10.1016/0006-8993(66)90029-1 5968925 10.1016/0006-8993(66)90029-1

[CR26] Issurin V (2005) Vibrations and their applications in sport. A review. J Sports Med Phys Fit 45(3):324–336 16230984

[CR27] Issurin VB, Liebermann DG, Tenenbaum G (1994) Effect of vibratory stimulation training on maximal force and flexibility. J Sports Sci 12(6):561–566. 10.1080/02640419408732206 7853452 10.1080/02640419408732206

[CR28] Jochum D, Konrad A, Lohmann LH, Cochrane D, Rittweger J, Vogel V, Warneke K (2025a) The merit of superimposed vibration for flexibility and passive stiffness: a systematic review with multilevel meta-analysis. J Sport Health Sci. 10.1016/j.jshs.2025.101033 40021055 10.1016/j.jshs.2025.101033 PMC12137185

[CR29] Jochum D, Vogel V, Warneke K (2025b) Acute effects of passive stretching with and without vibration on hip range of motion, temperature, and stiffness parameters in male elite athletes. J Funct Morphol Kinesiol 10(1):17 39846658 10.3390/jfmk10010017 PMC11755640

[CR30] Ko MG, Lee MM, Song CH (2020) A comparison of the effects of different stretching methods on flexibility, muscle activity, and pain threshold in ballet dancers; a preliminary randomized controlled trial. J Bodyw Mov Ther 24(4):354–360. 10.1016/j.jbmt.2020.06.019 33218533 10.1016/j.jbmt.2020.06.019

[CR31] Konrad A, Alizadeh S, Daneshjoo A, Anvar SH, Graham A, Zahiri A, Goudini R, Edwards C, Scharf C, Behm DG (2023) Chronic effects of stretching on range of motion with consideration of potential moderating variables: a systematic review with meta-analysis. J Sport Health Sci. 10.1016/j.jshs.2023.06.002 37301370 10.1016/j.jshs.2023.06.002 PMC10980866

[CR32] Konrad A, Budini F, Tilp M (2017a) Acute effects of constant torque and constant angle stretching on the muscle and tendon tissue properties. Eur J Appl Physiol 117(8):1649–1656. 10.1007/s00421-017-3654-5 28624851 10.1007/s00421-017-3654-5 PMC5506206

[CR34] Konrad A, Stafilidis S, Tilp M (2017b) Effects of acute static, ballistic, and PNF stretching exercise on the muscle and tendon tissue properties. Scand J Med Sci Sports 27(10):1070–1080. 10.1111/sms.12725 27367916 10.1111/sms.12725 PMC5479471

[CR33] Konrad A, Reiner MM, Thaller S, Tilp M (2019) The time course of muscle-tendon properties and function responses of a five-minute static stretching exercise. Eur J Sport Sci 19(9):1195–1203. 10.1080/17461391.2019.1580319 30821657 10.1080/17461391.2019.1580319 PMC6816483

[CR35] Kubo K, Kanehisa H, Fukunaga T (2002) Effect of stretching training on the viscoelastic properties of human tendon structures in vivo. J Appl Physiol 92(2):595–601. 10.1152/japplphysiol.00658.2001 11796669 10.1152/japplphysiol.00658.2001

[CR36] Lance J, Burke D, Andrews C (1973) The reflex effects of muscle vibration. In: Human reflexes, pathophysiology of motor systems, methodology of human reflexes, vol 3. Karger Publishers, pp 444–462

[CR37] Lewis AH, Cui AF, McDonald MF, Grandl J (2017) Transduction of repetitive mechanical stimuli by Piezo1 and Piezo2 Ion channels. Cell Rep 19(12):2572–2585. 10.1016/j.celrep.2017.05.079 28636944 10.1016/j.celrep.2017.05.079 PMC5646378

[CR38] Lundeberg T, Nordemar R, Ottoson D (1984) Pain alleviation by vibratory stimulation. Pain 20(1):25–44. 10.1016/0304-3959(84)90808-x 6333660 10.1016/0304-3959(84)90808-X

[CR39] Maeda N, Urabe Y, Tsutsumi S, Sakai S, Fujishita H, Kobayashi T, Asaeda M, Hirata K, Mikami Y, Kimura H (2017) The acute effects of static and cyclic stretching on muscle stiffness and hardness of medial gastrocnemius muscle. J Sports Sci Med 16(4):514–520 29238251 PMC5721181

[CR40] Mahieu NN, Cools A, De Wilde B, Boon M, Witvrouw E (2009) Effect of proprioceptive neuromuscular facilitation stretching on the plantar flexor muscle-tendon tissue properties. Scand J Med Sci Sports 19(4):553–560. 10.1111/j.1600-0838.2008.00815.x 18627559 10.1111/j.1600-0838.2008.00815.x

[CR41] Masud AA, Shen CL, Luk HY, Chyu MC (2022) Impact of local vibration training on neuromuscular activity, muscle cell, and muscle strength: a review. Crit Rev Biomed Eng 50(1):1–17. 10.1615/CritRevBiomedEng.2022041625 35997107 10.1615/CritRevBiomedEng.2022041625

[CR42] Mizuno T (2019) Combined effects of static stretching and electrical stimulation on joint range of motion and muscle strength. J Strength Cond Res 33(10):2694–2703. 10.1519/jsc.0000000000002260 29023326 10.1519/JSC.0000000000002260

[CR43] Mizuno T (2023) Combined static stretching and electrical muscle stimulation induce greater changes in range of motion, passive torque, and tendon displacement compared with static stretching. Sports (Basel). 10.3390/sports11010010 36668714 10.3390/sports11010010 PMC9864422

[CR44] Muanjai P, Haas C, Sies W, Mittag U, Zange J, Schönau E, Duran I, Kamandulis S, Rittweger J (2023) Effect of whole-body vibration frequency on muscle tensile state during graded plantar flexor isometric contractions. J Exerc Sci Fit 21(4):405–415. 10.1016/j.jesf.2023.10.003 37965131 10.1016/j.jesf.2023.10.003 PMC10641229

[CR45] Nakano J, Yamabayashi C, Scott A, Reid WD (2012) The effect of heat applied with stretch to increase range of motion: a systematic review. Phys Ther Sport 13(3):180–188. 10.1016/j.ptsp.2011.11.003 22814453 10.1016/j.ptsp.2011.11.003

[CR46] Nigg BM (1997) Impact forces in running. Curr Opin Orthop 8(6):43–47

[CR47] Nordin M, Hagbarth KE (1996) Effects of preceding movements and contractions on the tonic vibration reflex of human finger extensor muscles. Acta Physiol Scand 156(4):435–440. 10.1046/j.1365-201X.1996.465180000.x 8732248 10.1046/j.1365-201X.1996.465180000.x

[CR48] Oliveri DJ, Lynn K, Hong CZ (1989) Increased skin temperature after vibratory stimulation. Am J Phys Med Rehabil 68(2):81–85. 10.1097/00002060-198904000-00007 2930643 10.1097/00002060-198904000-00007

[CR49] Page P (2014) Beyond statistical significance: clinical interpretation of rehabilitation research literature. Int J Sports Phys Ther 9(5):726–736 25328834 PMC4197528

[CR50] Pantaleo T, Duranti R, Bellini F (1986) Effects of vibratory stimulation on muscular pain threshold and blink response in human subjects. Pain 24(2):239–250. 10.1016/0304-3959(86)90046-1 3960570 10.1016/0304-3959(86)90046-1

[CR51] Pereira BM, Magalhaes FA, Lacerda ACR, Andrade AGP, Peixoto GHD, Chagas MH (2020) Comparison of four local vibratory stimuli on mechanical and sensorial variables related to muscle-tendon unit response. Transl Sports Med 3(5):440–446. 10.1002/tsm2.150

[CR52] Rittweger J (2020) Manual of vibration exercise and vibration therapy. Springer, Berlin

[CR53] Ritzmann R, Gollhofer A, Kramer A (2013) The influence of vibration type, frequency, body position and additional load on the neuromuscular activity during whole body vibration. Eur J Appl Physiol 113(1):1–11. 10.1007/s00421-012-2402-0 22538279 10.1007/s00421-012-2402-0

[CR54] Robinson LR, Probyn L (2019) How much sciatic nerve does hip flexion require? Can J Neurol Sci 46(2):248–250. 10.1017/cjn.2018.378 30698119 10.1017/cjn.2018.378

[CR55] Rodrigues SA, Rabelo AS, Couto BP, Motta-Santos D, Drummond MDM, Gonçalves R, Silva RAD, Szmuchrowski LA (2017) Acute effects of single bout of stretching exercise and mechanical vibration in hamstring muscle. J Exerc Physiol Online 20(4):46–57

[CR56] Savadipour A, Palmer D, Ely EV, Collins KH, Garcia-Castorena JM, Harissa Z, Kim YS, Oestrich A, Qu F, Rashidi N, Guilak F (2023) The role of PIEZO ion channels in the musculoskeletal system. Am J Physiol Cell Physiol 324(3):C728-c740. 10.1152/ajpcell.00544.2022 36717101 10.1152/ajpcell.00544.2022 PMC10027092

[CR57] Skinner B, Dunn L, Moss R (2023) The acute effects of theragun™ percussive therapy on viscoelastic tissue dynamics and hamstring group range of motion. J Sports Sci Med 22(3):496–501. 10.52082/jssm.2023.496 37711710 10.52082/jssm.2023.496 PMC10499144

[CR58] Takeuchi K, Nakamura M, Fukaya T, Konrad A, Mizuno T (2023) Acute and long-term effects of static stretching on muscle-tendon unit stiffness: a systematic review and meta-analysis. J Sports Sci Med 22(3):465–475. 10.52082/jssm.2023.465 37711702 10.52082/jssm.2023.465 PMC10499138

[CR59] Taylor DC, Dalton JD Jr, Seaber AV, Garrett WE Jr (1990) Viscoelastic properties of muscle-tendon units. The biomechanical effects of stretching. Am J Sports Med 18(3):300–309. 10.1177/036354659001800314 2372082 10.1177/036354659001800314

[CR60] Vay L, Gu C, McNaughton PA (2010) Current perspectives on the modulation of thermo-TRP channels: new advances and therapeutic implications. Expert Rev Clin Pharmacol 3(5):687–704. 10.1586/ecp.10.41 22111750 10.1586/ecp.10.41

[CR61] Vay L, Gu C, McNaughton PA (2012) The thermo-TRP ion channel family: properties and therapeutic implications. Br J Pharmacol 165(4):787–801. 10.1111/j.1476-5381.2011.01601.x 21797839 10.1111/j.1476-5381.2011.01601.x PMC3312478

[CR62] Wang TG, Hsiao TY, Wang CL, Shau YW (2007) Resonance frequency in patellar tendon. Scand J Med Sci Sports 17(5):535–538. 10.1111/j.1600-0838.2006.00618.x 17316374 10.1111/j.1600-0838.2006.00618.x

[CR63] Warneke K, Plöschberger G, Lohmann LH, Lichtenstein E, Jochum D, Siegel SD, Zech A, Behm DG (2024a) Foam rolling and stretching do not provide superior acute flexibility and stiffness improvements compared to any other warm-up intervention: a systematic review with meta-analysis. J Sport Health Sci. 10.1016/j.jshs.2024.01.006 38244921 10.1016/j.jshs.2024.01.006 PMC11184403

[CR64] Warneke K, Rabitsch T, Dobert P, Wilke J (2024b) The effects of static and dynamic stretching on deep fascia stiffness: a randomized, controlled cross-over study. Eur J Appl Physiol 124(9):2809–2818. 10.1007/s00421-024-05495-2 38689040 10.1007/s00421-024-05495-2 PMC11365840

[CR65] Warren CG, Lehmann JF, Koblanski JN (1976) Heat and stretch procedures: an evaluation using rat tail tendon. Arch Phys Med Rehabil 57(3):122–126 1267581

[CR66] Weppler CH, Magnusson SP (2010) Increasing muscle extensibility: a matter of increasing length or modifying sensation? Phys Ther 90(3):438–449. 10.2522/ptj.20090012 20075147 10.2522/ptj.20090012

[CR67] Wilcox RR (2011) Introduction to robust estimation and hypothesis testing. Academic Press, Boca Raton

[CR68] Wilk KE, Macrina LC, Fleisig GS, Aune KT, Porterfield RA, Harker P, Evans TJ, Andrews JR (2015) Deficits in glenohumeral passive range of motion increase risk of shoulder injury in professional baseball pitchers: a prospective study. Am J Sports Med 43(10):2379–2385. 10.1177/0363546515594380 26272516 10.1177/0363546515594380

[CR69] Wilke J, Müller AL, Giesche F, Power G, Ahmedi H, Behm DG (2020) Acute effects of foam rolling on range of motion in healthy adults: a systematic review with multilevel meta-analysis. Sports Med 50(2):387–402. 10.1007/s40279-019-01205-7 31628662 10.1007/s40279-019-01205-7

[CR70] Willigenburg NW, Poolman RW (2023) The difference between statistical significance and clinical relevance. The case of minimal important change, non-inferiority trials, and smallest worthwhile effect. Injury 54(Suppl 5):110764. 10.1016/j.injury.2023.04.051 37923502 10.1016/j.injury.2023.04.051

