# Supplementary material for: Immediate effects of passive stretching and/or local vibration on ankle range of motion, calf muscle stiffness and passive torque: a randomized controlled cross-over trial
Source: Eur J Appl Physiol. 2025 Jun 12;125(11):3363–75. doi: 10.1007/s00421-025-05839-6 (PMC12528329; doi:10.1007/s00421-025-05839-6)
Supplement: Supplementary file 1 — Supplementary file1 (DOCX 28 KB) [file 421_2025_5839_MOESM1_ESM.docx]

# Supplemental Material

Supplemental Table 1 showing intraclass correlation coefficients with 95% confidence intervals for interday reliability.

|  | ICC (InterDay) | 95% CI |
| --- | --- | --- |
| ROM | 0.963 | 0.936 – 0.981 |
| Stiffness (0) | 0.766 | 0.595 – 0.876 |
| Stiffness (Max) | 0.941 | 0.897 – 0.969 |
| PRT (0) | 0.842 | 0.727 – 0.917 |
| PRT (ROMmax) | 0.948 | 0.910 – 0.972 |
| PPT (Max) | 0.949 | 0.911 – 0.973 |
